# The remarkable gymnastics of ORC

**DOI:** 10.7554/eLife.76475

**Published:** 2022-02-09

**Authors:** Bruce Stillman

**Affiliations:** 1 Cold Spring Harbor Laboratory Cold Spring Harbor United States

**Keywords:** helicase, origin licensing, replication, ORC, Mcm2-7, Cdt1, *S. cerevisiae*

## Abstract

As a cell prepares to divide, a molecular actor known as the Origin Recognition Complex makes intricate ATP-driven movements to recruit proteins required to duplicate DNA.

**Related research article** Gupta S, Friedman LJ, Gelles J, Bell SP. 2021. A helicase-tethered ORC flip enables bidirectional helicase loading. *eLife* **10**:e74282. doi: 10.7554/eLife.74282

Eukaryotic cells that are getting ready to multiply must first faithfully replicate the long DNA molecules that form their genome. This duplication process requires a carefully choregraphed ballet of molecular actors intervening at precisely the right time and place.

For example, in the yeast *Saccharomyces cerevisiae* – the best characterized DNA replication system to date in eukaryotes (Attali et al., 2021) – the first step consists of the Origin Recognition Complex (or ORC) attaching to specific ‘replication origin’ sequences in the genome (Bell and Stillman, 1992). This process requires binding of a tiny molecule known as ATP. Once in place, ORC can recruit other proteins to build a pre-replicative complex, which cells can activate when they are ready to duplicate their DNA.

The formation of this pre-replicative complex begins with ORC recruiting Cdc6, an enzyme that can also bind ATP. This ORC-Cdc6 structure loads two ring-shaped, hexamer complexes known as Mcm2-7 on to the DNA. Later, these Mcm2-7 hexamers form the functional core of two separate DNA helicases; these enzymes move in opposing directions from the replication origin to unwind the DNA helix, making it accessible to the DNA synthesis machinery (Attali et al., 2021; Noguchi et al., 2017). A gap in the barrel-shaped Mcm2-7 hexamers is kept open by the Cdt1 chaperone so that, when ORC-Cdc6 recruits the Mcm2-7-Cdt1 complex, it can feed double stranded DNA into this opening (Zhai et al., 2017; Yuan et al., 2020). The first Mcm2-7 hexamer then has double stranded DNA passing through its central channel (Yuan et al., 2017). While the identity of the components forming the pre-replicative complex are well known – ORC, Cdc6, Mcm2-7, Cdt1 – precisely how the second Mcm2-7 hexamer is loaded onto the origin DNA needed clarification.

For instance, it has been long known that ORC attaches to the replication origin in an oriented manner by recognizing a particular ‘A element’ motif; however, it can also bind, in the opposite orientation, to a ‘B2 element’ also present in the origin (Figure 1; Bell and Stillman, 1992). In fact, the assembly of the pre-replicative complex and the subsequent bidirectional DNA replication depend on these inverted ORC binding sites (Coster and Diffley, 2017). A logical conclusion was that two ORC molecules would load the two Mcm2-7 helicase hexamers in opposite directions, so that the two hexamers end up in their required head-to-head configuration. Yet, other data suggest that a single ORC could actually load both Mcm2-7 hexamers.

**Figure 1.**
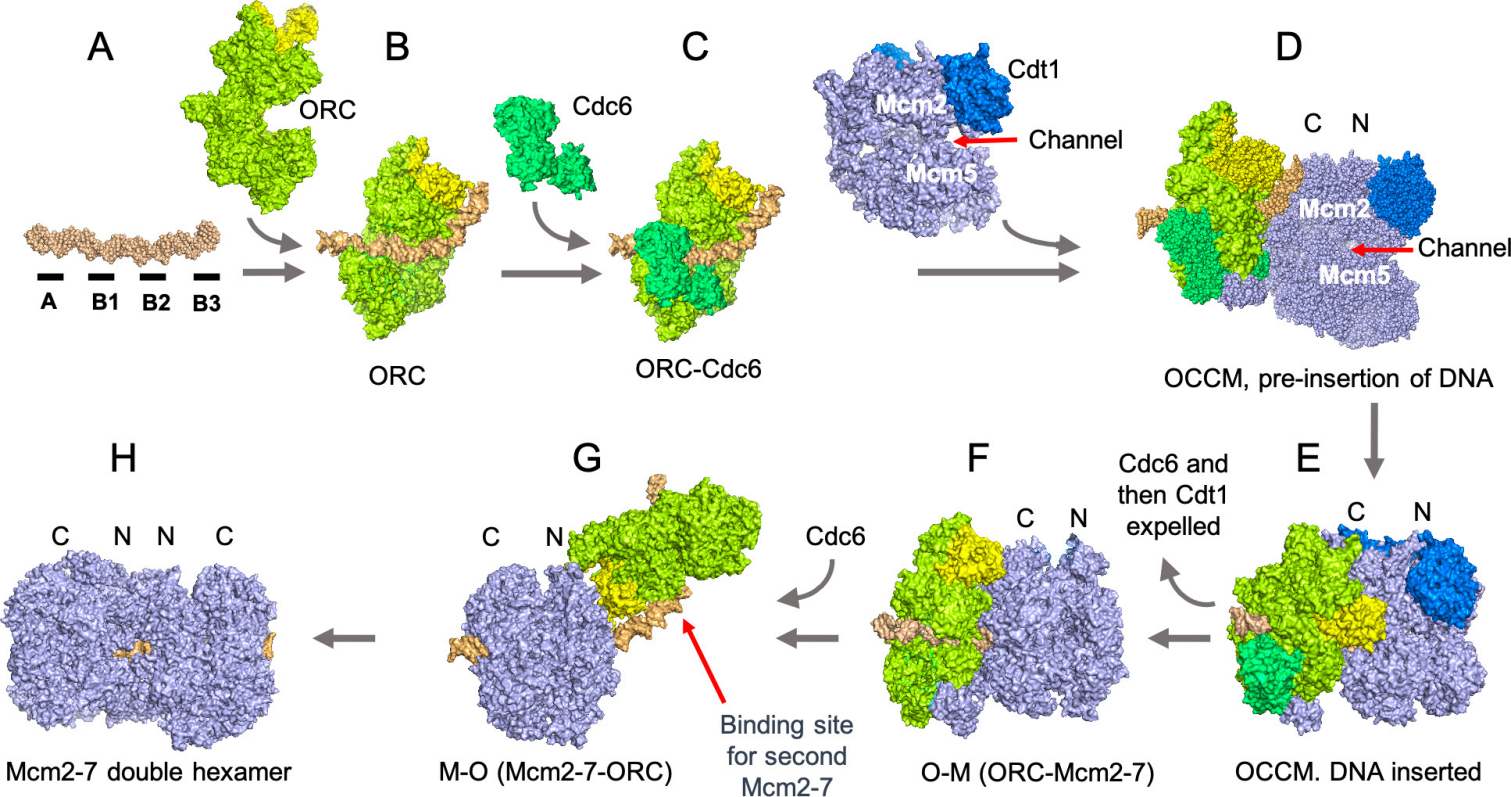
The assembly of the pre-replicative complex requires a seven-step process which involves ORC ‘flipping’ over the Mcm2-7 complex. (**A**) The genetic sequence which acts as the starting point for replication in *S. cerevisiae* (DNA segment in orange) consists of four elements (black segments), with the A and B2 elements binding ORC in opposite orientations. (**B**) ORC (lime green, Orc6 yellow) first binds to the A and B1 elements. (**C**) ORC recruits Cdc6 (green). (**D**) ORC-Cdc6 recruits the Cdt1-Mcm2-7 complex (Cdt1, blue and Mcm2-7 light blue; Mcm2 and Mcm5 are parts of the six proteins in the complex) so that the DNA is aligned to a channel in the Mcm2-7 hexamer. The Mcm2-7 complex is oriented based on the structure of the protein components; with a C-terminus (or extremity; Mcm-C or ‘C’) at one end, and an N-terminus (Mcm-N or ‘N’) at the other. At this stage, Mcm-C binds ORC-Cdc6. (**E**) The double stranded DNA is inserted into the channel between the Mcm2 and Mcm5 subunits in the Mcm2-7 hexamer and the hexamer is partially closed, to create an intermediary known as the OCCM. (**F**) ATP hydrolysis by the Mcm2-7 expels the first Cdc6 and then Cdt1 to create the OM complex. (**G**) ORC flips to the other side of Mcm2-7 and presumably binds to the B2 element, creating the MO complex. As a result, the ORC subunit Orc6 is now orientated towards Mcm2-7-N. (**H**) ORC can now recruit a second Cdc6, creating a binding site for a second Cdt1-Mcm2-7 complex that is loaded in an opposite orientation to the first Mcm2-7. The Mcm2-7 double hexamer, possibly with ORC still bound to the DNA, establishes the pre-replicative complex that is a precursor for the activation of two enzymes that will unwind the DNA helix when the cell is ready to divide.

Image credit: Molecular structures taken and adapted from the RCSB protein database, deposits 5BK4, 5V8F, 5XF8, 6RQC and 6WGG (Noguchi et al., 2017; Miller et al., 2019; Yuan et al., 2020; Yuan et al., 2017; Zhai et al., 2017).

Examining ‘intermediary’ structures that form as the pre-replicative complex assembles can help to shed light into the mechanisms involved. Such investigations have become possible since scientists have been able to assemble pre-replicative complexes in the laboratory using purified proteins, allowing single-molecule microscopy approaches such as cryo-EM to be paired with genetic and biochemical manipulations (Evrin et al., 2009; Remus et al., 2009; Frigola et al., 2013; Miller et al., 2019; Noguchi et al., 2017; Yuan et al., 2017). As a result, studies have revealed the existence of an intermediate structure known as the OCCM (formed of ORC-Cdc6-Cdt1-Mcm2-7), in which double stranded DNA passes through the middle of the ORC-Cdc6 and Cdt1-Mcm2-7 ring-shaped complexes (Yuan et al., 2017). Other work has highlighted additional intermediate structures consisting of the Mcm2-7-ORC complex bound to DNA in either a MO (Mcm2-7- ORC) or OM (ORC- Mcm2-7) orientation (Miller et al., 2019; Figure 1).

In this study, counting of individual molecules revealed that 74% of the complexes had one ORC, either at the A element or the B2 element, whereas 26% had two. This suggested that loading of the Mcm2-7 double hexamer could occur with either one or two ORCs. Now, in eLife, Stephen Bell and first author Shalini Gupta from the Massachusetts Institute of Technology – with colleagues Jeff Gelles and Larry Friedman at Brandeis University – report new insights that address how one ORC is able to load a second Mcm2-7 hexamer (Gupta et al., 2021).

To conduct their experiments, the team enlisted a replication origin which contained a double stranded DNA segment tethered in a chamber. They labelled various segments of ORC, Cdt1 and Mcm2-7 with different fluorescent compounds; a special type of microscopy called TIRF was then used to track these tagged proteins and how they interact as the pre-replicative complex assembles. The data clearly showed that a single ORC molecule can load two Mcm2-7 hexamers in a head-to-head manner.

To do so, ORC first recruits a Cdc6 protein, and together they load a Cdt1-Mcm2-7 complex – forming the previously identified OCCM complex. ATP is then broken down to release energy, which expels Cdt1 and Cdc6, leaving the MO intermediate bound to the origin. ORC then adds a second Cdc6 which loads another Cdt1-Mcm2-7 hexamer in the opposite direction on the DNA, creating the double Mcm2-7 hexamers in their head-to-head configuration (Figure 1).

The remarkable observation is that ORC switches from one side to the other of the first Mcm2-7 hexamer, presumably jumping between binding to the A and B2 elements. The ‘flip’ creates a new intermediate structure consistent with the MO complex observed previously (Miller et al., 2019; Figure 1). ORC’s impressive gymnastic move is possible because the first Mcm2-7 hexamer breaks down ATP, providing the required energy for the complex to slide along the DNA strand, exposing the B2 element. This allows ORC to then flip orientation, and to load the second Mcm2-7 hexamer in the right position. It remains to be determined how this occurs in other eukaryotic cells, most of which do not have specific genetic sequences similar to the A or B2 elements in their replication origins.

Large ATP-driven molecular movements have been known for some time – a classic example being the motor proteins kinesin or dynein, which can use ATP to change conformation and shuttle molecules by ‘walking’ on the cell’s internal skeleton (Gennerich and Vale, 2009). The results by Gupta et al. provide another, remarkably elegant example of the diverse and intricate ATP-powered feats which keep cells ticking.
